# "The Hospital" Nursing Mirror

**Published:** 1901-02-09

**Authors:** 


**"The Hospital" Nursing Mirror.**

Being the Nursing Section of "The Hospital."

[Contributions for this Section of "The Hospital" should be addressed to the Editor, "The Hospital" Nursing Mirror, 28 & 29 Southampton Street Strand, London, W.C.]

### Queen Alexandra and the Nursing Vocation.

The universal sorrow for the death of Queen Victoria is tempered by universal gratitude for the fact that the gracious lady who has so long been known and esteemed as Princess of Wales is now Queen Alexandra. To nurses in particular her accession to the dignity of Queen Consort is especially welcome. The active interest she has always manifested in their well-being and the sympathy she has been so quick to show them whenever an opening offered, have endeared her to them in a remarkable manner. They need no assurance that in the more exalted position which in the Providence of God she has been called upon to occupy she will maintain the attitude of solicitude for their elevation, and concern for their welfare, which she has invariably observed ever since the welcome of the British nation was voiced in Tennyson's splendid verse :—

> Welcome her, welcome the land's desire,
> The sea-king's daughter as happy as fair.
> Blissful bride of a blissful heir,
> Bride of the heir of the kings of the sea.

Queen Alexandra has, in a variety of ways, on many occasions, given practical proof of her desire to help those whose true mission in the world is to help others at the most trying time of life. Her kindness of heart, the gentleness which is one of her distinguishing traits, her unaffected piety, would naturally prompt her to render any assistance she could to the Divine work of succouring the sick, and she has quietly availed herself of every possible opportunity to identify herself with it. This has been demonstrated again and again during the course of the campaign in South Africa. It will be fresh in the memory of our readers how, little more than a year ago, she determined to send out twelve highly-trained ladies, to be known at the seat of the war as the Princess of Wales's Nurses, and how characteristically she received them before their departure, investing them herself with her own badge, and speaking to each the well-chosen words which are always at her command. In her anxiety to be of service to our wounded soldiers, she resolved to supply them with a hospital ship of her own, which she completely equipped, and at her instance a large number of them were tended on their return in a luxurious private hospital. She regularly visited the vessel on its arrival in port, and conversed with the nurses whom she had chosen, while those engaged in her hospital had more than once the pleasure of answering her thoughtful inquiries about the progress of their patients. The gold cross which she presented the other day to the sister who was in charge of her hospital ship during its voyages will be a cherished souvenir; and in acknowledging the substantial sum which the nurses voluntarily contributed through our columns to her War Fund, she used language which conveyed to every subscriber a sense of personal appreciation. She has watched with close attention the arduous labours of the army sisters, and has learnt with deep regret that, while some died at the post of duty, the physical resources of others have been overtaxed. Among the manifold evidences of interest she takes in the nursing vocation is the existence of the Alexandra Nurses, who form an important adjunct of the Soldiers and Sailors' Families Association, and who will soon have the first of their own "Alexandra" Homes. But Queen Alexandra's great work in the nursing world has, of course, been the unique and valuable aid she has afforded to the movement with which her name is yet more inseparably associated—the Royal National Pension Fund for Nurses. Admirable as are the objects of the Fund, they would not so soon have commended themselves to several thousands of nurses if the Princess of Wales had not been its active and sagacious, as well as its highly honoured, President. When, on July 4th, 1890, the President received at Marlborough House the first thousand of the nurses who had joined the Fund, and presented them with their certificates, the incident was described in The Hospital as "the most interesting event in the history of the world in connection with workers among the sick." But even then, perhaps, its full significance was not realised; nor was it anticipated that it would be the means of knitting together the Throne and the nursing vocation in a bond never, we believe, likely to be broken. It was, indeed, the first occasion on which representatives of any philanthropic institution had been invited to Marlborough House, but since then the members of the Royal National Pension Fund have been three times the favoured guests of the present King and Queen of England. The reception in 1891 was followed by an event which cemented the tie between the President and those who by virtue of the amulet they wear, have the privilege of being called her nurses; for while the screen containing their photographs which was presented to her as a birthday gift on December 12th, 1891, is among her treasured possessions, her autograph letter of thanks is one of their most precious belongings. July 28th, 1895, and July 21st, 1899, are marked in the reddest of letters in the calendar of the five thousand members of the Pension Fund who were presented by the President with certificates at the third and fourth receptions. The value of these delightful functions it is impossible to over-estimate. How directly they affect the growth of the Pension Fund may be gathered from the circumstance that, after each of them there has always been a large increase in the number of policy-holders—a fresh stage of development. But they have a worth beyond this. The simple but cordial hospitality, the tact with which the President puts her guests at their ease, the pleasant word, the bright smile which she has for all, convert admiration into enthusiasm, and loyal respect into loving reverence. That these sentiments will be strengthened and intensified it needs no prophet to foresee; and from the heart of every nurse in Christendom will go up to Heaven the earnest prayer that Queen Alexandra may long be spared to adorn the Court of which she is both the fairest ornament and the sweetest personality.

### Notes on News from the Nursing World.

### MISS FLORENCE NIGHTINGALE AND THE PASSING OF THE QUEEN.

OUR readers will learn with interest that the Funeral Procession through the streets of London on Saturday was witnessed by Miss Florence Nightingale from the window of her house. Of all the vast concourse which assembled on that never-to-be-forgotten occasion, none we may be quite sure, was more in sympathy with the spirit that pervaded all classes than the great nurse who had special cause to honour and esteem the great Queen whom she survives.

### QUEEN VICTORIA'S JUBILEE INSTITUTE FOR NURSES.

THE Queen's nurses working in England, Scotland, Ireland and Wales, united to send a wreath to Osborne in memory of their beloved Queen, the founder of the institute. The wreath was an oval about three feet by four, of dark violets with sprays of lighter Neapolitan violets and clusters of lilies of the valley; it bore the following inscription: "With deepest sorrow and most devoted loyalty, from the Queen's Nurses throughout England, Scotland, Ireland and Wales."

### IRISH NURSES AND QUEEN VICTORIA.

SOME striking floral tributes were sent over from Ireland for the funeral of Queen Victoria. That of the matrons and nurses of the Adelaide Hospital, who were received by the Queen last year, took the form of an Irish harp composed of violets. The harp was 3½ feet in height. The ground work was composed of violets, and the crown of the same flowers of a darker hue—Neapolitan violets. On the right-hand side, fixed to the frame, was a fine spray of Roman hyacinths, and on the left a cluster of lily of the valley. Another effective decorative device was a cluster at the bottom of the harp, composed of Harrisii lilies, lily of the valley, and white tulips, with foliage of cocos palm, croton leaves, and asparagus ferns intertwined. The golden chords were intertwined with shamrocks, and a big bow of heliotrope, perfectly matching the violets and contrasting pleasantly with the white of the other flowers, further enhanced the effect. The card accompanying the offering bore this inscription: "In sorrowing and dutiful remembrance from the matrons and nurses of Dublin." Mourning is being worn by many of the Dublin nurses: a black band on the left arm by the Adelaide Hospital staff, and by many other nurses black silk badges with "V.I." in silver, worn on both indoor and outdoor uniform.

### NURSES AT THE PASSING OF THE QUEEN.

THE sisters and nurses of St. Mary's Hospital were invited by Dr. Wilfred Harris to go on Saturday morning to see the Funeral Procession from his house in Oxford Terrace. A platform was erected with seats for twenty in the garden in front of the house. The matron willingly accepted the invitation, and by 9.45 the nurses were all in their places. They had a capital view of the proceedings - there was nothing, not even a lamp-post, to mar the line of vision. The recollection of the grandeur of that solemn sight will be of lifelong duration to one and all. While they are grateful to Dr. Harris for his kindness in giving them such an unexpected pleasure, they also appreciate the self-sacrifice of their co-workers amongst the nurses who worked right cheerily to enable them to get off up to time. Several of the probationers and night nurses went to the Nurses' Home in Cambridge Place, where they had a good bird's-eye view of the pageant. There was a small stand in Grosvenor Gardens containing a number of nurses.

### LORD KITCHENER AND THE SISTERS.

ANYONE visiting the Yeomanry Hospital at Pretoria will, writes one of our correspondents, feel inclined to agree with Lord Kitchener, who recently, when visiting the wards, expressed to the sisters a desire to "come in for a few days' rest." The hospital was formerly the residence of a wealthy Uitlander and is situated in one of the suburbs of Pretoria. Since the sad death of Prince Christian Victor, whose gentleness and patience made a great impression, there have been many fatal cases of enteric—it appears to attack the younger men. Many of the deaths lately have been those of patients under 25.

### "B.P.'s" NURSES.

THE vacancies for sisters in General Baden Powell's new hospital at Pretoria are to be filled up. The ladies appointed will receive salaries of £120 per annum, equivalent to £80 in England, a uniform of khaki, and dark green capes with green slouch hats and feathers.

### SAD DEATH OF AN ARMY NURSE.

THE death of Miss Emmie Owen in South Africa is announced. She was accidentally drowned at Germiston. Miss Owen, who was trained at the Royal Infirmary, Derby, only left England last August for service in South Africa. She was the younger daughter of the Rev. J. Stanley Owen, Vicar of St. Alkmund, Derby, who formerly held curacies in London, one at Bermondsey and the other at Stepney. Mr. Owen is also chaplain to the Derby Borough Asylum. The sad fate of his daughter, who was very anxious to be sent out to nurse the sick and wounded soldiers, has caused much regret to a large circle of relatives and friends.

### QUEEN VICTORIA'S NURSES IN SCOTLAND.

PROGRESS continues to be made by the Scottish branch of Queen Victoria's Jubilee Institute for Nurses. During the last three months of 1900, the Scottish Council were responsible for forty-three probationers at various stages of hospital and district training, as well as for a permanent staff of seven nurses. New branches were formed at Clydesdale, Roberton, and North Ronaldshay; and six nurses who completed their training were engaged by affiliated associations.

### MARRIAGE OF A "BART'S" SISTER.

THE wedding of Miss Rosamond Rolleston, which was to have taken place on Saturday, but was postponed in consequence of the Queen's funeral, was quietly celebrated on Monday, at Christ Church, Lancaster Gate. The service was fully choral, and of course the guests were all in mourning. The old Oxford friends of the bride were present in force, but the intended reception afterwards was not held. Miss Rolleston is the daughter of the learned Professor Rolleston, formerly Professor of Physiology at Oxford. She was trained at St. Bartholomew's, and won the silver medal in 1892. After acting as night superintendent for some months, she was appointed sister of Elizabeth Ward. While holding this post—which affords very varied experience—she contributed several interesting articles to the columns of "THE HOSPITAL" NURSING

MIRROR, and she also took a prominent part in the social life of the nurses at St. Bartholomew's, in the debating society, and on the committee of the nurses' library. At the commencement of the war Miss Rolleston volunteered for the A.N.S.R., and went out to South Africa on the ss. *Guelph* in February 1900. Very soon after the League of St. Bartholomew's Hospital Nurses was inaugurated, and "Sister Elizabeth's" letters to the matron found a prominent place in the first number of the *League News*, which is published half-yearly. The bridegroom—Dr. J. A. Hayward—is a "Bart's" man; and after qualifying, in 1890, was house physician for Sir Dyce Duckworth, who is one of the visiting physicians for Elizabeth Ward at St. Bartholomew's. He is now in practice at Wimbledon.

### THE CRISIS AT PORTSMOUTH INFIRMARY.

THERE has been yet another resignation at Portsmouth Workhouse Infirmary. Moreover, not only has a charge nurse followed the example of the matron and assistant matron, but twenty-two nurses have signed a document which shows that the sympathy of a considerable proportion of the staff is with the retiring officials. In this letter it is stated that Miss Court had endeared herself to all. Her position, however, it proceeds, had been a very trying one, and the unfortunate want of co-operation on the part of some of her colleagues had not lessened the difficulties of her office as matron. As members of the nursing staff, the signatories of the communication could not but deplore the resignation of their two principal colleagues, as it was felt that the loss of their services would seriously hamper the work of the establishment. In conclusion, the signatories suggested that the interests of the infirmary would be best served by the resignations being allowed to be withdrawn. At a meeting of the Infirmary Committee this communication was discussed, some of the guardians condemning it and others approving, Miss Kingswell in particular among the latter. She observed that Miss Court had got on very well indeed with the nurses with the exception of one or two, while the patients never had anything but praise for her. She considered that the nurses had a right to express their opinion, and, personally, she regretted that Miss Court was leaving the institution. An attempt on the part of Mr. Kennedy, a guardian, who described the communication as "a piece of sheer impudence," to ignore it was unsuccessful, and it was determined to acknowledge the letter. An inquiry is being made by a special committee into the cause of the crisis, and the issue of their report may, perhaps, affect the situation.

### A NURSING HOME FOR DARLASTON.

IT has been decided to establish a nursing home at Darlaston, and try it for three years with two nurses. The estimated cost is from £180 to £200 a year, and promises of annual subscriptions amounting to £165 for each year have been received. This does not include the subscriptions of the working men or friendly societies, who are expected to contribute, and it may therefore be said that the movement starts extremely well. In such a town as Darlaston there is probably scope for more than two nurses; but the support already secured is an earnest of the desire of the residents to make ample provision for the needs of the sick.

### WORDS OF WISDOM FROM WHITSTABLE.

A TIMELY protest has been made by Mr. Edward Austin, of Whitstable, against the introduction of the *odium theologicum* into nursing matters. Because the committee of the Whitstable District Nursing Association have appointed a nurse who is supposed to be a High Churchwoman a certain number of persons in the town have denounced both the committee and the nurse. Mr. Austin, who declares that he is the staunchest Protestant in Whitstable, severely condemns their attitude, and says:—

> What we need in Whitstable is a good nurse, and a good nurse should not be disqualified, even though she be utterly at variance with one's theological views. As for working with Ritualists, I hail any opportunity of working with any man for a good cause, be his creed what it may. The gulf is wide enough already; why should we unnecessarily widen it? We often fight because we do not understand each other, and I for one would rather heal than wound.

These are words of wisdom, which should have the effect of silencing those other Protestants who are sufficiently narrow-minded and unjust to charge, as Mr. Austin puts it, "a useful body of ladies with making the care of the body a blind for turning from their faith those whom they nurse."

### A RETROGRESSIVE PROPOSAL.

MR. WILLIAM AUSTIN, the clerk to the Luton Board of Guardians, has been lecturing on "Luton's 20th century needs" with reference to Poor Law administration. Having made the admirable statement that "one of the greatest blessings now brought within the reach of the poor in many districts is the ministration of trained nurses," he went on to say that the supply is far from meeting the demand.

> Why it should be thought that a nurse can only be ranked as a certificated nurse if she has received her training in an institution having a resident medical officer I cannot see. I think workhouse infirmaries with over 200 inmates and all hospitals having a staff of trained nurses should have the privilege of granting certificates to probationer nurses who have proved themselves efficient after a reasonable period of training in such institutions.

As the official position occupied by Mr. Austin may invest these remarks with importance in the eyes of some people, we may observe that the policy he advocates is distinctly retrogressive. If it were adopted, we should inevitably not only have many standards of training, but a number of so-called trained nurses very little removed in professional efficiency from the old Sairey Gamp.

### NURSES AND VISITORS.

LAST week we reported a meeting of advocates of a scheme for visiting the sick in the London hospitals, at which one hospital official was present, who said, "Sisters and nurses and children wait, and the visitors don't come." We can understand that the little people at the Sick Hospital for Children like to see ladies in pretty clothes; but that sisters and nurses generally are desirous for an influx of visitors in hospitals we are sure is not the case. On the contrary, their feeling is, in the main, utterly opposed to a campaign of the kind contemplated by the excellent persons who met in conclave and chattered at Eaton Place, alike in their own interests and in those of the patients. It is obvious that the frequent intrusion of visitors would seriously interfere with their duties, and any innovation that would have this result must necessarily be to the detriment of the sick and suffering under their charge. The preposterous talk about "dreary wards" shows almost as marked a lack of acquaintance with the existing conditions of hospital life as the observation of a speaker who alluded to workhouse infirmary nurses as belonging to "the commonest description of pauper" proves gross ignorance of the character of infirmary nurses. There is room for much improvement in respect to nursing in workhouse infirmaries, but even in the most badly managed institutions the nurses are not paupers at all, while in those of a better class they are, of course, trained ladies—probably ladies of superior culture and education to some of the amiable ladies with abundance of leisure who want to invade the hospitals in the "latest thing in hats."

### A MYSTERY AT NEWTON ABBOT.

THE Visiting Committee of the Newton Abbot Board of Guardians, having inquired into the alleged non-performance of her duties on the part of one of the nurses, and consulted the medical officer, the master, superintendent nurse, and all the nurses, reluctantly recommended the board to request her to send in her resignation. At a meeting of the guardians the committee's recommendation was carried by 17 votes to 14, and it transpired that the committee had spent several hours in investigating the affair, and came to the conclusion that "the only way out of the difficulty" was to ask the nurse to resign. It was also stated that the nurse had been told that she could resign if she liked, but that she had not done so. One of the members of the committee asked that particulars of the charge against the nurse for which she was called on to resign should be placed before the full board; but this was refused, though the name of the nurse had been already made public. So that practically the board adopted the report without knowing the nature of the charges. The nurse must have had some reason for not accepting the notice to resign, and if the committee did not intend to explain to the other guardians the reasons for their action they should also have refrained from making the nurse's name public talk. In common justice she should have had the opportunity of laying her defence before the full board, especially in view of the fact that since the meeting half the nursing staff—who append their names to their statement—have openly denied helping to influence the committee to ask for the nurse's resignation. They add: "We all feel sorry that Nurse —— is to be made the scapegoat, because in November last, she, as senior nurse, tried to get matters righted, and spoke for us all."

### YORK WORKHOUSE INFIRMARY.

THE chairman of York Board of Guardians has been criticised by some of his colleagues for speaking out respecting the management of affairs by the house committee. At the last meeting he said, "First there was the disturbance with the nurses, then the difficulties with the officials, the inquiry that followed, and the bad management revealed by that inquiry," and he added that "those who were anxious to conduct the affairs of the union in a proper way could not be pleased with these reports." With regard to the statement that the first disturbance was about the nurses, we are informed that when the superintendent nurse made her first complaint—which was a very grave one—the guardians spoke of the unpleasantness and friction that had then been going on for some time. The superintendent nurse declared that she was not upheld by the board, and that between the opposition of the master and matron and the insolence of some of the nurses she found herself in anything but a bed of roses. The position thus described is an impossible one; but since the chairman has insisted upon the need of amendment, it is permissible to hope that the nursing and other difficulties to which he refers, will be overcome to the advantage alike of the ratepayers of York, the patients, and the infirmary staff.

### ASHTON NURSING ASSOCIATION.

IT is a matter for regret that the report of the Ashton Sick Nursing Association is not, from a financial point of view, satisfactory. But though it had been hinted that a decrease in the nursing staff was in contemplation, we are glad to learn that this backward step in a town where population is steadily increasing is not likely to be adopted. On the contrary, the committee, convinced that the work of the nurses is appreciated on all sides, will go forward with the confidence that more generous support will be given the more the necessity for it is known. No doubt the collections in the mills and the workshops which last year amounted to £79, can be still further increased, as well as the regular subscriptions. The Ashton Board of Guardians already contribute, and the society appears to be admirably managed.

### DRESSING GOWNS IN WORKHOUSE INFIRMARIES.

THE aged inmates of the Exeter City Workhouse have reason to be grateful to the guardians who stood up to defend their proposal to provide half-a-dozen dressing gowns for use in the infirmary. The proposal met with a little opposition, a member of the Board arguing that not 1 per cent. of the ratepaying community have the privilege of such garments; but as the gowns are intended to replace those now in use when the patients in the infirmary leave their beds, such an objection was just a little capricious. We have never yet heard of the Poor Law institution where the aged poor were made too comfortable, and we believe that nurses who have to work in such places would be only too glad if the provision of dressing gowns became more general, for in their absence they find that the lack of such garments is frequently rather awkward. Nowadays the workhouse infirmaries are, as a rule, filled with people who have come there to end their lives, and any little comfort that can be added is much appreciated by those who have seen better days. Whether suitable articles can be obtained at less than 12s. 6d. is another matter, but the Board were almost unanimous in adopting the recommendation of the Labour and Supplies Committee.

### THE GENERAL HOSPITAL, BARBADOS.

WE hear that it has not been found possible to secure another head nurse for the General Hospital, Barbados. Perhaps if the managers were able to improve the terms which have been quoted in our columns, and to make things generally more comfortable than the two last head nurses found them, there would be no lack of applications for the post.

### SHORT ITEMS.

NURSING Sister Edith Ward, who has been seriously ill at Pretoria, is reported out of danger.—During January, district nursing associations at the following places have been affiliated to Queen Victoria's Jubilee Institute for Nurses:—Hemel Hempstead (Herts.), Horsforth (Yorks.), Matlock (Derbyshire), Totnes (Devon), and Broughton (N. Wales).—A Sessional Lecture will be given on Thursday, February 14th, by Mrs. H. J. Tennant, ex-H.M. Superintending Inspector of Factories, on "Nurses and the Industrial Laws," at the rooms of the Royal British Nurses' Association, 10 Orchard Street, Oxford Street, W., at 5.30 P.M.

### Lectures to Midwifery Nurses.

By ROBERT JARDINE, M.D., &c., Physician to the Glasgow Maternity Hospital. Examiner in Midwifery to the University of Glasgow, &c.

### SOME BLADDER COMPLICATIONS OF THE PUERPERIUM.

AFTER difficult labours we sometimes find incontinence of urine. Pressure upon the neck of the bladder has caused paralysis of the sphincter, so that the woman is no longer able to retain her urine. It constantly dribbles away from her. As a rule the sphincter will regain its power in a few days, and the dribbling cease.

Here, as during pregnancy, the overflow from a distended bladder may be mistaken for incontinence. There is another condition which may be mistaken for incontinence—viz., urine flowing from an opening in the bladder wall, what is called a vesico-vaginal fistula. We shall discuss this condition a little later on.

The patient we have before us is a multipara with a contracted pelvis. Before she was admitted forceps had been tried without avail. After admission we applied axis traction forceps, and tried to deliver her in the Walcher position, but failed. We might have dragged the head through, but it was quite evident the child would be dead and the maternal parts dangerously crushed. We therefore did craniotomy. A symphysiotomy might have been done here, and I probably would have done the operation if no attempt at delivery had been made before admission. The true conjugate is about 3½ inches. The day after the operation I was told that she had incontinence. I ordered a catheter to be passed, and about 2½ pints of urine were drawn off. It was really not a case of incontinence, but of retention with an overflow. Since then she has, however, had incontinence, which is gradually improving. To-day we are going to examine her thoroughly, as she is going out soon. It is just possible that there may be a small vesico-vaginal fistula here as there has been sloughing of the bruised vaginal wall, so we shall test the bladder to see if there is a hole in its base. The way to do this is to fill the bladder with a coloured fluid. Milk, which of course has been boiled to sterilise it, is what we are going to use. Now that we have injected a pint and drawn back the perineum so as to expose the anterior vaginal wall you see none of the milk comes into the vagina, so there is no fistula. If a fistula is very minute it is sometimes difficult to find it.

This is an interesting case for you to see as it illustrates two conditions, viz., retention and then afterwards incontinence. In a few days more no doubt she will have perfect control over her bladder.

Retention of urine is the commonest bladder complication you will meet with in the puerperium. It is most frequent in primiparæ who have had difficult labours, especially if the perineum has been torn and stitched. The same condition arises in operations on the perineum or rectum irrespective of labour. In multiparæ it is also sometimes seen from the same cause. In every puerperal case the patient should be encouraged to pass her urine a few hours after delivery. If she cannot do so the first time you may wait a few hours and let her try again, but don't wait more than ten hours. To assist her apply to the vulva a warm cloth wrung out of lysol solution and place her on her hands and knees. The lower part of the binder should, of course, be unpinned. Some warm water should be placed in the utensil, as the steam which rises soothes the parts. If she fails pass the catheter by sight in the way already described. Generally one passage of the catheter will be sufficient, but you occasionally have to repeat it, especially if the bladder has been allowed to become very much distended. Over-distension paralyses it, and it may be some days before it regains its tone.

If you are attending cases as a midwife you should make a habit of always asking the patient on your first visit after the labour if she has passed urine, and if she has not done so make her try to, and if she fails pass the catheter. If you do not attend to this she may not tell you, and if you are not seeing her again until the next day and she has retention she will suffer a great deal and considerable harm may be done. I make it an invariable practice to ask on my first visit if the urine has been passed. I have had more than one bad case of inflammation of the bladder to treat in patients who had not been asked this simple question by the medical attendants. They had not liked to tell they were unable to pass urine until the condition had become unbearable. With a monthly nurse in charge of course there should be no risk of this occurring.

*Cystitis, or Inflammation of the Bladder.*—This condition may exist during pregnancy as well as during the puerperium. During the puerperium it is apt to arise in cases where there has been retention of urine necessitating the use of the catheter. It is almost sure to arise if a dirty catheter is used or some of the lochial discharge carried into the bladder on a clean catheter. Hence the importance of absolute cleanliness of the catheter and the patient's vulva.

The patient will have frequency of micturition and a burning pain in the bladder. The urine may become very septic and be loaded with pus. It will be thick and muddy and deposit a heavy sediment. The patient's temperature and pulse will rise.

The treatment as far as a nurse is concerned will be to give the patient plenty of diluent drinks such as milk and soda or barley water. In bad cases the bladder should be washed out once or twice daily with warm boracic lotion. Alkalies and sedatives are given by the mouth and various drugs such as boracic acid to act on the urine.

In bad cases the inflammation may spread up the ureters and affect the kidneys, so it is not a condition to be trifled with.

*Vesico-vaginal fistula.*—By this term is meant an opening between the vagina and bladder. The urine flows through the opening so you have a constant dribbling from the vagina. It may be mistaken for incontinence or vice versa.

Vesico-vaginal fistulæ are now not nearly so common as they were many years ago. They are caused by the anterior vaginal wall being injured by prolonged and severe pressure during labour. The parts are so injured that sloughing takes place and when the slough separates the bladder is opened into. The slough will not separate under a week or more so that the urine will not begin to dribble away immediately after labour. This distinguishes it from incontinence, as the latter usually begins immediately after labour. It is exceedingly rare for a fistula to be formed at the time of labour, unless from accidental perforation of the bladder with an instrument. If you find urine beginning to flow continuously from the vagina during the second week of the puerperium, you may be pretty sure there is a fistula.

I have said fistulæ are not so common as they used to be. The reason of this is that no woman is now allowed to remain for many hours in the second stage of labour until the parts are seriously injured. At first forceps were blamed, as fistulæ formed in many cases in which they were used. In those days forceps were only applied after very many hours, when the woman was exhausted. It was not the forceps which caused the mischief, but the prolonged pressure of the head. The forceps were not used soon enough. The timely use of forceps is the great preventative of fistulæ. A midwife must bear this in mind, and send for assistance early when there is delay in the second stage.

The condition is usually easy of diagnosis if the fistula is of any size, as you can easily feel it and see it with a speculum. If it is very small it can only be found by filling the bladder with a coloured fluid in the way I showed you to-day.

A patient so affected will suffer great distress. Her urine constantly dribbles away and the external parts soon become sodden and excoriated.

You must try to keep her as dry as possible, and dust the external parts with zinc or other powder, or smear them with zinc ointment.

Small fistulæ will close of themselves, but an operation is generally necessary. The operation should not be done until the parts are quite healthy. Six or eight weeks after the confinement the edges of the opening should be rawed and the parts stitched very accurately together. In very large fistulæ, where the base of the bladder is practically all destroyed, it is sometimes necessary to close the vagina completely. The upper part of the vagina along with the damaged bladder then forms a receptacle for the urine, and the woman is relieved from the constant dribbling.

Sometimes the fistula is not between the bladder and vagina but higher up, between the bladder and the cervix or uterus. The opening cannot then be seen, but the coloured fluid will run from the external os. There are also one or two other rare varieties, but they are too complicated for nurses to understand.

### A Royal Nat. P. Fund Nurse's Version of the National Anthem.

God save our King and Queen,
Long may they live and reign,
God bless them both.
Send them victorious,
Happy and glorious,
Long to reign over us,
God bless them both.

Thy choicest gifts in store
On them be pleased to pour,
God bless them both.
May they defend our laws,
And ever give us cause
To sing with heart and voice,
God bless them both.

### Appointments.

Keighley Union Infirmary.—Miss Sarah Eleanor Mather has been appointed matron and superintendent nurse. She was trained at Chorlton Union Hospitals, Witherington, near Manchester, and has since been superintendent nurse at Rochdale Union Infirmary, superintendent of a private nurses' home in Rochdale, and superintendent nurse at Prestwich Union Hospital, Crumpsall, Manchester.

### Minor Appointments.

Middlesex Workhouse Infirmary.— Miss E. A. Cawood has been appointed charge nurse. She was trained at Middlesbrough Workhouse Infirmary.

### Where to Go.

The Dowdeswell Galleries.—Mr. Walter Tyndale is showing a charming collection of water-colour drawings. The subjects are for the most part Italian, scenes in Venice, Rome, Perugia and other cities being depicted.

### The East Preston Guardians and the Superintendent Nurse.

On Tuesday Mr. J. S. Davy, Poor Law inspector, and Dr. A. Fuller, one of the medical officers of the Local Government Board, held an inquiry at East Preston Workhouse, Brighton, with a view of ascertaining whether the Local Government Board would be justified in giving their assent to the dismissal of Miss M. Rogers from the position of superintendent nurse.

### The Evidence of Miss Rogers.

Miss Rogers first stated her case, and said she was appointed superintendent nurse at East Preston in September, 1899. Before that she was matron and superintendent nurse at East Grinstead for four years. She held the certificate of the London Obstetric Society for midwifery, and that of the London Homœopathic for general work. The condition of affairs on her arrival at East Preston was very bad throughout. There were practically no medical appliances in the infirmary. There were, for instance, no scissors or forceps; there were no bandages in store except those that were being used; there was only one bed cradle, and that was broken; and there were then consumptive patients in the ward. The nightstools were mostly of iron, and smelt very badly; and the bowls were of papier-mâché, very old, and used for all washing purposes. The only medicines she had were cough mixture, aperients, and diarrhœa mixture. The stock of clothing and underlinen was very short, and it had been short until about twelve weeks ago. Since she had been at East Preston inmates had worn their flannel vests for five and six weeks, neckerchiefs for six and seven weeks, and one man wore his worsted stockings for ten weeks. She had spoken to the matron time after time with reference to the supply of more clothing, but for various reasons it was not forthcoming. She also complained to the committee about the lack of necessary medicines, &c. With the exception of some boracic acid and some strappings none of the things she asked for had ever been supplied, and she still felt the inconvenience and want of them. She referred to several cases in detail as tending to show that sick patients had been unduly kept either in the workhouse or receiving wards, and that patients under her charge had been allowed to leave the house without her being consulted. Some seven or eight months after she came to East Preston, Mr. Davy paid a visit to the infirmary, and she afterwards read in the papers that the condition of the nursing had very much improved. When she came there the discipline was very bad: it was no uncommon thing to find the nurses quarrelling and swearing, and even throwing clothing at each other. She also considered the staff was inadequate, and in consequence of her representation to the Nursing Committee they appointed one additional nurse. She thought they wanted two more assistant nurses, and also paid women to do the scrubbing and cleaning of the infirmaries. She had not seen a really hot dinner served in the infirmary since she had been there. On November 6th last she was requested to submit her resignation, but neither the Guardians nor the Nursing Committee had ever made any complaint with reference to her administration of the infirmary, except that on one occasion one of the Guardians remarked, "If I may speak my mind the infirmary will never be right till you are gone."

### Cross-examination of Miss Rogers.

In the course of cross-examination, Miss Rogers said she was still unconscious of having acted in any way so as to justify the Board in taking the steps they had; and it was because of this that she had declined to send in her resignation. A few days ago the master refused to let an inmate named Cager go with witness to see her solicitor's representative. She left East Grinstead in consequence of the compulsory resignation of the master on the ground of ill-health, and in addition to the highest of testimonials the Guardians gave her a gratuity of £50 for loss of office. She complained that she had been hampered in her duties by the matron; she complained of the conduct of the master; she complained of the insolence of a nurse and other persons; and she also accused the Nursing Committee of having vented on her their annoyance at having to appoint her by direction of the Local Government Board as superintendent nurse; but she did not complain of the medical officer. She did not know that there had been any friction between her and the other officials, but she believed that they had tried to prevent her from carrying out her duties. She had not sent the committee an amended list of medicines and other necessaries, because she was never asked for one. She regarded the absence of labels as serious: an unlabelled bottle was a very serious thing when bottles contained medicines. She had no feeling against the master and matron, and she denied that she had ever said in the hearing of patients that she would "make it hot" for them. In conclusion she explained at length the nature of some visits she had received on legal business.

### The Medical Officer's Views.

Mr. Charles A. Lapthorne, medical officer to the Workhouse, said that having heard the complaints and suggestions of the superintendent nurse, in his opinion the Nursing Committee had given attention to her reasonable requests as far as they could. He also considered the present nursing staff to be quite adequate, although, at some times, more help might be given them in the way of the hard work, like scrubbing. It was, however, very difficult to get people in that place at a minute's notice to do the work. In his opinion the receiving ward was a suitable place for its purpose. Miss Rogers had complained to him of the ward attendants being withdrawn from the sick wards when they were badly needed there. She had also complained of the want of sufficient linen, and he had spoken to the matron about it. All his requests had always been complied with. It was preferable, of course, to have separate sheets for the maternity ward, but Nurse Rogers had never made a request to him for a fresh supply of linen for this ward. It had been brought to his notice that there had been considerable friction lately in the infirmary, and he wrote to the Guardians some time ago, and suggested that they might frame some rules as the hours when the nurses must be in, and the Guardians did so. He was of opinion that it was imperative and necessary that something should be done to improve things in the infirmary. With regard to the general administration of the infirmary, he had never heard any complaints from the patients about the superintendent nurse being away from the wards for any undue length of time. The superintendent nurse would have a better opportunity of judging as to how the other nurses performed their duties than he had. On the whole, there had been a decided improvement in the state of things at the infirmary since Miss Rogers had been in charge. He thought that the superintendent nurse might have done more of the physical work herself, but with this exception she had performed her duties in a satisfactory manner.

### Other Witnesses.

Miss Gates, of Worthing, who was in the Maternity Ward in October last, confirmed some of the complaints made by Miss Rogers. Miss Hitch, who has had ten years' experience as nurse, and who has been at East Preston since December 4th, 1899, said there was nothing at all for the nurses' use at that time—neither dressing trays, forceps, syringes, nor, indeed, anything they wanted to use. It was also very difficult to obtain clean clothes for the inmates. Nurse Byrne adhered to the statements she had made in her communications to the Board. With reference to the case of a child who died from whooping cough, the superintendent nurse bullied her in the hearing of the other patients, and told her if she had exerted herself a little more the child would not have died. When Nurse Rogers had found fault with her she had taken her own part and defended herself. The matron said she could not believe that the patients had been compelled to wear stockings and other things for the time stated by Miss Rogers; and the master testified that there had been considerable friction between the nurses and superintendent. All the nurses but one had complained.

### The Nursing Committee's Defence.

Mr. Michael King, the Chairman of the Nursing Committee of the Board of Guardians, said he recollected the terms of the superintendent nurse's engagement: she was to assist the other nurses from time to time as required. All her requests for articles or other things had been complied with by the committee as far as they possibly could. Besides applying for all these articles the superintendent nurse also made repeated complaints, and this was really the source of all the trouble. They thought the superintendent had been the cause of all the friction, and they had come to the conclusion that no improvement was possible so long as she remained. They accordingly recommended the Board to ask for her resignation, which they did. He was away at the time himself, but he believed the resolution of the Board was unanimous. Personally he had had no reason to change his opinion since then. As to there being any feeling on the part of the Nursing Committee against the appointment of a superintendent nurse, he could only say that, although some of them thought it was not advisable, yet when it was decided they must appoint one the committee had sought, by every fair means, to carry the decision into effect and make the change a success. The Guardians did not suspend the superintendent nurse at first, because they expected to get the reply of the Local Government Board every day but by the next meeting they got tired of waiting, and decided on her suspension. A prompt rejoinder then came from the Local Government Board that they had no right to do this, and she had since been allowed to continue her duties. He was a frequent visitor to the infirmaries, and he considered they had always been kept scrupulously clean. When Miss Rogers made her complaints against the other officials they heard their replies and came to their decision in her absence. There was not the slightest complaint against her in her capacity as nurse, but they did object, among other things, to her seeking to have sole control over the infirmaries. The committee had dealt with the most urgent of Miss Rogers' requirements at once, but many of the things could have been supplied from stock had she applied to the master or matron. She did not say that she was applying to the committee because she could not get them from the matron. The inquiry then closed, having lasted for over seven hours.

### The Scene on the Solent.

A correspondent sends us the following:—

"We waited upon the shore on a little piece of rising ground to the right of the old fort, nearly opposite Ryde. The haze hanging over the sea lifted gradually as the day wore on, and one by one the huge men-of-war loomed out into distinctness. In the sunshine it was warm as a June day, and except where in the shade the frost still gleamed white, and for a touch of north in the air, we forgot that it was yet winter. A wonderful hush was over everything, hardly a sound except that of the tiny waves lapping upon the beach. For miles there lay the battleships, looking more and more imposing as the mist cleared off. Up and down between the motionless ships darted a torpedo-boat, and now and again a steam-launch swept quickly by. The first gun boomed out at last, and then each man-of-war in turn was wrapped in the smoke from its cannon. It was strange to see white rings separate themselves from the cloud of smoke, hover in the air, and slowly melt away. The guns roared on and on. At last a tiny yacht became visible in the distance, gliding slowly between the rows of battleships. On and on she came, till we could see through our glasses all the details of the burden she was carrying. Her decks were bare except for those few black figures steadfastly keeping watch and ward. Under the canopy on deck there lay beneath a standard of white satin all that remained of the beloved dead. On the satin standard were placed the crown, sceptre, and sphere, which never more would be used by the dead Queen. The little yacht passed on, carrying its burthen and sorrowing mourners down the pathway of the sea. Behind followed Royal yachts bearing the King and many relatives. As they swept by the music of funeral marches from the warships mingled with the roar of the guns. The last to salute was the old *Victory*, who had so often paid homage to the Queen in her lifetime. The sun was sinking low in the sky when the yachts came to anchor in the bay, and then we turned to go. The scene was indescribably beautiful, the sky flooded crimson and gold, and the sea reflecting the golden light. The guns had ceased firing, silence reigned over all, and as we looked back yet once more we knew that the beauty and majesty of the scene would never be forgotten."

### Echoes from the Outside World.

### AN OPEN LETTER TO A HOSPITAL NURSE.

ALTHOUGH the last sad rite is over, and Queen Victoria has been laid to rest beside the husband whom she loved so dearly, it is almost impossible to write of any other subject whilst the recent sorrowful days are so fresh in one's mind. The very remembrance of the glorious funeral pageant seems well-nigh overshadowed by the simple fact that now even the mortal remains of our beloved Sovereign are taken from us, and we begin the world afresh, with a new ruler at the helm. As I passed Buckingham Palace on Friday and saw the men strewing with gravel the roadway which was to be the scene of "The Queen's Last Ride," I could not help reflecting upon the many times when she had driven up to the Palace and her carriage had borne her through the gates amidst the plaudits of the multitude. But I knew that when she passed on the morrow there would be no turning in; 'that wonderful coach" would bear her on whilst her people in reverent silence with tearful faces would watch her progress.

---

IN the precincts of the residence itself there were few signs of mourning, except the down-drawn white blinds and the black bands on the arms of the liveried servants. Even the wreaths of laurel and evergreen which were hung upon many houses and upon all the other lamp-posts of the route were absent along the Mall and in the Park, the Office of Works having refused to the ladies' committee who undertook the collection and distribution of these wreaths permission to hang them there. Miss Etta Close and other ladies were the originators of the scheme, and by Wednesday night their appeal had been answered by the arrival of the 800 required. All classes of people contributed them, peers and members of the House of Commons, school children, costers, artisans, the dwellers in towns and the toilers in villages. But when once the supply started it seemed inexhaustible, and before Saturday over 3,000 wreaths had been received, showing the pleasure it gave to many to render any service, however small, in Her Majesty's name. Most were utilised though a very few have been sent, I am told, to two of the hospitals.

---

THE 2nd of February began at a very early hour for many who wished to see the pageant. The last trains which came from the country the previous evening brought in a crowd of humble folks who could not afford the phenomenal prices of the hotels and apartments, and who spent the night wandering about the streets or in the Park. The cold was intense, and those who had provided themselves with an "Etna" and appliances for tea-making were much envied by the less fortunate. The trains which came about five or six also poured forth fresh sightseers, and the pedestrians arrived even earlier. We slept in town so as to avoid setting forth in the darkness; but rest was an impossibility in the neighbourhood of Buckingham Palace Road. All night the workmen erected stands and hung up drapery by the light of flaring gas jets. At four o'clock the first battalion of soldiers came galloping by in the strange lurid light, and by six there was a thin line of patient men, women, and children stretching all along the pavement. Those who joined our party later told of twenty people in each railway carriage, frequent detentions on the line, and inexorable policemen who refused to allow seatholders to cross the line of route at any point. I even heard of some who though able to gaze at their goal from afar, never succeeded either in reaching their seats or in seeing the procession from elsewhere. They were just forced to remain in the streets till it was time for a train to take them back tired and disappointed.

OF course, from our position, we did not see the procession in its extreme length, because before it began to move the head of it stood at Devonshire House, Piccadilly, and the blue-jackets of the Royal Navy, who were about the centre of the cortége at the entrance to Buckingham Palace Road. About a quarter past ten the military attachés to the foreign embassies assembled close to Grosvenor Gardens. Most of them were unknown to the populace, but with them was one whom everyone knew at once, and as Earl Roberts, holding his Field Marshal's bâton in his hand, and riding on a most beautiful Arab charger, came in sight, a spontaneous and irrepressible cheer rose from the crowd. It was hushed almost immediately, as if the people felt ashamed of having been betrayed into even a semblance of rejoicing, but all eyes were fixed upon the upright lithe figure seated so closely upon his horse. I could not help being struck with the glow of health upon the cheery old face, and even the sadness did not mar the serene expression habitual to him whom we love to designate as "Bobs." Then, after a further wait, a white flag was waved by a mounted horseman as the sign for the advance and we knew that the last glimpse of our Queen was coming. How slowly the men marched! They scarcely seemed to lift their feet from the ground; how sadly they bowed their heads, and how mournful and soul-stirring was the "Funeral March" as the band, with drums concealed in crape, passed between the troops with their arms reversed! Not a sound could be heard in the crowd; the tramp, tramp of the soldiers' feet vibrated on the quiet air, and it appeared as if all London were holding its breath as the glittering bier came into view. The eight cream ponies were almost hidden by the scarlet and gold trappings, the only touch of mourning being violet bands and streamers on the horses' heads. The gun carriage showed its khaki wheels, but all else was concealed by the white satin pall, its four corners aflame with the emblazoned arms of England and with the colour of the silken Standard thrown across it. At the head, upon a pale-blue cushion, reposed the Crown; at the feet lay the orb and the sceptre; and every insignia of Royalty was there, as I know it was fitting that it should be. But I found it strangely difficult to realise that amidst this pomp lay the mortal remains of Queen Victoria. Before my eyes, again, rose the picture of June, 1897, a noble-looking old lady, clad in quiet black, with only one touch of white amidst her sombre headgear, the simplest person of all around her, acknowledging with tears the plaudits of the gaily-dressed people who had come to do her honour.

---

AND as I gazed both visions passed, and I knew that a fresh page of history had been turned, for there behind the coffin rode His Majesty the King, Edward VII. He looked very regal in his Field Marshal's uniform, though it was easy to see the traces upon his usually genial face of all he had gone through the last ten days. It was hard to accord him no welcome, as he moved by upon the Queen's favourite mare, but we knew he would rather have it so, and the gaze of all travelled on to that other Monarch who has of late secured such a firm footing in the hearts of the English people—the German Kaiser. His pallor was extreme, and yet there was a kindliness and a sympathy in his expression which I failed to remember when he was in London some few summers ago. The Duke of Connaught, though his moustache is very white, is the same brave-looking prince we remember of yore, and special interest attached to the King of Portugal, a big florid man, the gay colour of whose uniform accorded well with his appearance; the King of Greece, refined and rather sad-looking, closely resembling his brother the Crown Prince of Denmark; the Crown Prince of Germany, growing much like his father, and the youthful Duke of Saxe-Coburg Gotha, who, though so young and small, bore himself nobly, and evidently felt much the fresh sorrow which has entered his life. Of the ladies inside the carriages we saw nothing. Only the King of the Belgians, with his long grey beard, looked out and bowed slightly ever and anon. Then the non-commissioned soldiers of the German Army came by, and for Londoners the funeral pageant of the greatest Queen the world has ever seen was over.

### Examination Questions for Nurses.

### EMERGENCY NURSES.

### Result of January Competition.

The question was as follows: "In the case of a man severely kicked in the abdomen by a horse, what are the chief dangers to be apprehended, what symptoms would you expect, and what course of treatment should you pursue, supposing medical assistance not to be at once obtainable?"

### The First Prize.

This month (speaking broadly) the papers have been very good, though there are some deplorable failures, of which more anon. "Nurse Maud" is selected to receive the first prize because her answer is excellent and very simple. She sums up the situation succinctly in saying the dangers to be apprehended are "injury to the internal organs, collapse, hæmorrhage, and subsequent peritonitis." It would have been perhaps better to put collapse first, for some constitutions are so susceptible that serious shock and collapse occur without any extensive injury; but this fact does not come much under the notice of any but those of long experience in accident wards. Her idea of an improvised dressing is good; external wounds, however, are seldom present in these cases. A horse's hoof is a very broad and comparatively smooth weapon of injury, and this fact, combined with the thickness of the sufferer's clothes and the elasticity of the abdomen, prevents external wounds. Very frequently there is even no visible bruise.

### The Second Prize.

"Nurse Dorothea" wins the second prize. Her answer is good, but too diffuse and discursive. If it is ever her good fortune to be sister in charge of large wards, and to instruct probationers, she will learn the value of condensing her information, presenting the salient points forcibly to the notice of her pupils, and not confusing them with side issues. She understands her subject well, but she presents it badly to the reader. An unprofessional woman of common-sense reading Nurse Maud's paper would have an excellent idea of how to proceed in such an emergency, but not so with the seeker after knowledge who reads Nurse Dorothea's. I hope she will pardon these strictures and profit by them; it is a sign that hers is a "hopeful case," which inspires us to apply the sharp remedy of severe criticism.

### Honourable Mention.

Three nurses have gained honourable mention: "Nurse Ida," "A Registered Nurse," and "Shamrock." N.B. "A Registered Nurse" is not a sufficiently distinctive pseudonym; the same may be said of "Mary," "Lily," "Rose," &c. . . . The choice of such well-known names gives trouble and sometimes causes disappointment. Another point to be noted is the necessity of writing an answer straight along the paper, not in groups of sentences under different headings. Such answers cannot be published owing to lack of space, and so disqualify their writers from gaining prizes. I have such an answer now from Nurse Annie, though in no case would she have been successful. Study simplicity in all things I beg of you.

### First Prize.

In the case of a man being severely kicked in the abdomen by a horse the chief dangers to be apprehended would be injury to the internal organs, collapse, hæmorrhage, and subsequent peritonitis. The symptoms I should expect would be pain, fainting and coldness, vomiting, weak pulse, and perhaps unconsciousness.

While awaiting medical aid I should place the patient in the recumbent position (in bed if possible), with the head low; put a pillow beneath the knees to relax the abdominal muscles, and apply warmth to the body and extremities. If the patient is very collapsed and there are no signs of internal hæmorrhage, I should inject 3 or 4 oz. of warm milk, or beef tea with 1 oz. of brandy, per rectum, giving nothing by mouth but a few sips of hot water or strong coffee. Should the pain be severe I should try to relieve it by the application of hot flannels to the abdomen, keeping the weight of the bedclothes from the patient's abdomen by an improvised cradle. If there are symptoms of internal hæmorrhage, indicated by extreme pallor, fainting, weak or nearly imperceptible pulse, and anxiety of expression, I should raise the foot of the couch or bedstead, apply ice or cloths wrung out in cold water to the abdomen, and keep up the application of heat to the extremities. If the abdomen is wounded, and no antiseptic dressing was at hand, I should boil a piece of soft linen and apply it warm, covering it with cotton wool if available; if not, with thick folds of clean linen and a bandage.

Nurse Maud.

### Second Prize.

*Chief Dangers to be Apprehended.*

(α) Laceration of the peritoneum, setting up peritonitis.

(β) Injury or rupture of a large blood-vessel, with extravasation of blood into peritoneal cavity, setting up peritonitis or causing death from collapse.

(γ) Rupture of part of the viscera. In the case of liver or spleen, severe hæmorrhage into peritoneal cavity; in the case of stomach or intestines, escape of their contents into peritoneal cavity, setting up very rapid and fatal peritonitis; in the case of kidney or bladder, extravasation of urine.

(δ) Rupture of the rectum, with effusion of blood followed by suppuration and abscess.

(ε) In the case of a wound accompanying the injury there is the danger of its penetrating to the peritoneal cavity, and protrusion of the viscera; or in non-penetrating wounds there may be complications from effusion of blood, and always the danger of suppuration. These wounds also are sometimes followed by ventral hernia.

*Symptoms to be Expected.*

Shock, greater or less according to seriousness of injury. Collapse. In peritonitis an anxious countenance, yawning, rapid thin pulse, vomiting, local or general pain in abdomen, with complications of hæmorrhage, coldness, blanched wet skin, no vomiting and no pains (sometimes). In ruptured stomach or intestines vomiting contents of stomach, then blood and fæcal matter; blood passing by anus; intense local pain.

External hæmorrhage from wound.

*Course of Treatment to be Pursued supposing Medical Advice not to be at once Obtainable.*

Prepare bed for patient, using hair or wool mattress, and water-bed pillow for back, if obtainable, bottom sheet, mackintosh and drawsheet, one pillow for head, hot blankets next to patient. Place him in bed as quickly as possible, lying on his back, undress, and put on hot flannel shirt. Support knees with pillow and put hot-water bottles or bricks to extremities. Raise foot of bed on blocks or chairs if much collapsed, or there is any sign of hæmorrhage. Cleanse and draw wound as soon as possible. In the absence of antiseptic lotions and dressings use sterilised water and sterilised old linen, pack well with wool or dry sterilised old linen, and fasten on with towel for binder. If there is no external wound hot fomentations may be applied to region of pain, or ice (if obtainable) in case of hæmorrhage. No food by mouth except very small quantities of milk and water (ʒj. doses) or milk and ice (if obtainable). Keep patient as quiet and as still as possible, and prepare quietly what the doctor will need when he comes.

Nurse Dorothea.

### Defects in Many Papers.

There is very frequently a tendency in young and inexperienced nurses to attempt too much. More than one nurse in answering the above question makes the very serious statement that if the symptom of retention of urine was present, she should pass a catheter. This is a grave error. In the first place it is very frequently a matter of great difficulty to pass a catheter on a male patient even for a doctor, and false passages are very easily made. Secondly, it is quite unnecessary for any nurse to take such an extreme step in any civilised place, for even in the country a doctor can usually be found in a few hours, and there would be no immediate need of drawing off the urine. Thirdly, though no nurse worthy the name would hesitate to do anything for her patients' good where necessity arose, it is well to remember *his* feelings and not to distress a helpless man by such action unless imperatively necessary.

One nurse gives, as the immediate result of a severe kick, nearly all the physical ills that flesh is heir to, including cancer, pericarditis, dropsy, aneurysm, and bed-sores. I fear that unthinking young woman will never make a nurse.

In no case has any competitor mentioned in the symptoms of internal hæmorrhage, the peculiar grey tone which spreads over the face in such cases. It is very striking and not observable under any other conditions.

THE EXAMINER.

THE QUESTION FOR FEBRUARY.

What symptoms would lead you to suspect incipient hip disease in a child?

RULES.

The competition is open to all. Answers must not exceed 500 words, and be written on one side of the paper only. The pseudonym, as well as the proper name and address, must be written on the *same* paper, and *not on a separate sheet*. Papers may be sent in for fifteen days only from the day of the publication of the question. All illustrations strictly prohibited. Failure to comply with these rules will disqualify the candidate for competition. Prizes will be awarded for the two best answers. Papers to be sent to "The Editor," with "Examination" written on the left-hand corner of the envelope.

N.B.—The decision of the examiners is final, and no correspondence on the subject can be entertained.

In addition to two prizes honourable mention cards will be awarded to those who have sent in exceptionally good papers.

### Lecture to Nurses at the City Orthopædic Hospital.

ON Friday last the first of this series of lectures to nurses for this season was given by Mr. Noble Smith, senior surgeon to the hospital, who lectured upon "The Nursing of Orthopædic Cases." He referred to the important distinction which ought to be made between inflammatory disease of the spine and joints and non-inflammatory deformity. The inflammatory cases, which generally meant tubercular disease, required the greatest care and as much rest of the diseased parts as possible, and a great deal depended upon the nurse whether this necessary rest was carried out or not. In disease of the spine—often called "angular curvature" or Pott's disease—children were often kept lying down in bed, but unless they were most thoroughly strapped down their spines could not be kept at rest, because every movement disturbed the spine. At the City Orthopædic Hospital a fixation splint was applied which was much more effectual. Often the patients were kept in bed or on a prone couch even in addition to the use of this splint, but when they had improved they were allowed to walk about. When a patient was strapped to the bedstead the bedstead became the splint, and a very cumbrous one it was.

The lecturer then described a method of washing these patients without disturbing the diseased parts.

The child was first to be placed flat on its back, thoroughly supported by the mattress and by pillows. Then the straps of the instrument could be unfastened and the front of the body exposed. That part could next be carefully washed, afterwards the straps were to be accurately buckled up again, and the child turned gently on to its stomach and chest, when the straps were to be again undone and the back washed. The apparatus was not to be pulled away, but just lifted aside like the lid of a box, so that it could be replaced and buckled up again without disturbing the patient in the slightest degree.

Mr. Noble Smith gave a great many other details regarding the nursing of such cases—details which ought to be useful to all nurses having to attend to children.

The subject "The Nursing of Orthopædic Cases" will be continued on Friday, February 15th.

### Everybody's Opinion.

### RULES IN PRIVATE NURSING.

"A WATCHER" wishes to thank "E. J." for his kindly sympathetic words.

### MOURNING FOR QUEEN VICTORIA.

"VERITATE VINCIMUS" writes: I am sure that all readers of your paper will—after the paragraph in this week's NURSING MIRROR respecting the mourning for nurses, and the suggestions of the Lord Chamberlain—be interested to hear of the loyalty shown by the nursing staff of the Greenwich Infirmary. Out of deep respect and love for her late gracious Majesty Queen Victoria, they have worn mourning since January 28th. On their bright blue uniforms the matron, the sisters, staffs, and probationers all wear an inch-wide black silk band, which is fastened on the upper portion of the left sleeve; many added to the white waist-belts collars, and cuffs fastenings of black solitaires. When the Queen was known to be dangerously ill it was quite pathetic to hear the patients anxiously ask after the state of her health, "Was she any better?" "What was the latest news of her?" quite putting to one side their own serious illness; and later, if they might wear their own pieces of black ribbon in memory of the great and noble lady, who always took such a deep and sympathetic interest in their welfare. One loyal small sufferer, to whom a medallion of the late Queen had been given for bearing so bravely a long and painful surgical dressing, spent all the pennies that had taken her so long to save, in black ribbon for her hair. We are very loyal, and we loved our great and noble Queen; still, we do not forget that now our loyalty is due to a King, and he is her son.

### "B.-P." AND THE NURSES.

"A. N. S. R." writes: I should like to send a word of protest against the remarks of the *Daily Telegraph* correspondent quoted in your issue of December 15th just to hand. I am one of the staff of the hospital to which General Baden-Powell's "picturesque study of a pretty girl, made still more pretty by a khaki blouse and a B.-P. hat with a green puggaree and a jay's wing" was sent (with the idea of securing volunteers), and it may still be viewed here. I should like to state that when it was handed round the dinner-table for inspection, not a single Army Nursing Reserve Sister was "crazy to join," nor had, nor has, any intention of joining General Baden-Powell's staff of nurses. As far as nine years' nursing experience teaches, a "pretty kit" is not inducement enough to undertake nursing in Africa or any other place; and it would need the name of no General, however popular, to draw volunteers for duties of far greater risk and sacrifice than attendance on the sick police. Familiarity breeds contempt, we know; but it is rather hard to think that the correspondent in question, if he met any sisters of the Army Nursing Reserve, should have formed so low an opinion of their motives and work as to put into writing such a story of a large class of working women.

### BOTH SIDES OF THE QUESTION.

"A NURSE WHO LIKES FAIR PLAY" writes: It sounds selfish, as crudely written, almost the first thing a nurse asks of a private patient "Where am I to have my meals?" Doubtless that "almost the first thing" means after some chat, and all arrangements had been made, that query was put. Can the public understand the small amount of enjoyment nurses get out of life? There was a little excitement attached to dining *table d'hôte*; probably the question was asked innocently, if thoughtlessly. Why should a nurse be immaculate? She is only human, and will err sometimes. People in private life are, more often than not, selfish, tactless, and bad-tempered, yet if a nurse once asks a foolish question "shame" is called on her head from the whole medical and social world. The same people may sit down to a dinner of nine or ten courses, or a luncheon of four or five, yet if nurse only inquires where she is to dine, people say "victuals and drink" is all she thinks of. I trust the man who was paltry enough to make such a petty fuss over a woman, especially one trying to earn her living, is thoroughly ashamed of himself; such mean spite is not worthy of a man, leaving a gentleman out of the question. Does he realise the harm and ruin he has made for that nurse in her profession? Thank

Heaven! there are some gentlemen left who understand a nurse can be a gentlewoman as good as their sisters, mothers, or wives, whom they would not dream of telling to dine with their upper servants.

### NURSES' UNIFORMS.

"M. E. R." writes: Your readers may be interested in the following little incident which well illustrates the abuse practised in the wearing of a nurse's uniform. One evening about midnight into the casualty room of one of our large Midland hospitals a woman was led, or rather supported, between two policemen. She was dressed in the ordinary uniform of a trained nurse. The house-physician soon discovered that she was suffering from the effects of alcohol, and she was warned that the battery and other stringent measures would be taken unless she could control herself. Subsequently she was placed in a casualty ward for the night, as she was quite unfit to return home. She described herself as a trained nurse. On inquiry next day it was found that she had acted in the capacity of wardmaid in two hospitals, and on these facts she traded, and considered herself entitled to wear a nurse's uniform. The sight of such a woman overcome by drink would necessarily bring great discredit on the nursing profession. Then, again, we have the lady who likes to see her children's nurse decked out in uniform—trained or untrained it matters little. Instead of a safeguard to nurses, as it should be in many large towns, it is quite the reverse, and I have heard many a nurse say that she does not care to be seen in it now. If a regulation uniform (outdoor only, of course) could be decided upon, to be worn only by the certificated, every trained nurse would rejoice, and once more the nurse's cloak would be hailed with the respect it deserves. Surely this could be done if an effort were made to induce Government to take the matter in hand.

### SISTERS AND PROBATIONERS.

"SENIOR" writes: I agree with C. F. Y. that the giving up of nursing by probationers is often the result of the unjust bullying of an irritable ward-sister. I know of one case in which the sister was prejudiced beforehand against a new probationer who was coming to her ward. Matron had shown the sister one of the references, in which a well-meaning clergyman had given full particulars of the probationer's education. Sister read it, then said: "Send her to me. I'll take it out of her: these learned ladies are usually no good for work." In these days of educated gentlewomen as nurses, one fails to see the force of her remark. Be that as it may, this poor, unsuspecting probationer spent a most miserable two months. As C. F. Y. says, "harried" and called "slow" and "stupid," only in much stronger terms. She certainly would have given in had it not been for affording an opportunity of saying, "I told you so" to those of her friends who did not wish her to train, and who had prophesied that "a month would disenchant her." The work was not too hard, but of course she made the usual probationer's mistakes, and each one was held up and sneered at, and repeated with additions to every sister in the place. However, matron put her in a different ward, and she stayed on; when her turn came to go once more in the first ward sister was very different. Now the probationer has finished her training and likes her work very much. But this shows how many probationers, like her, have almost given up in despair "because sister is so horrid" and would probably not have courage to try anywhere else.

Miss LOUISA TWINING writes: I am glad to find that attention is being drawn to this matter of which I wrote lately, giving facts which had been within my knowledge for some years. But I feared to make my letter too long, and I now ask to be allowed to add one more remark, which I omitted, but which I consider is of equal importance. I allude to the work required in the training of probationers—one of their chief complaints, I may say. Many of these young women complain of the large amount of time given to merely manual work, such as cleaning (even sometimes the inside of windows) lockers and all brass-work, nearly the whole day being thus occupied in some hospitals, rendering the efficient learning of nursing, for which many have paid heavy fees, well-nigh impossible. Now, I would ask, Can this be necessary for a nurse's training during the whole three years? Surely any girl with average common sense and ability would be able to learn such housemaid's work and duties in three months, if not in less time, and why should they spend years in doing it, except for the one object of saving expense to the hospitals? Of course it is necessary that they should know how such work should be done, but I ask again, Why are such monotonous and laborious duties to occupy so large a proportion of the valuable time of training? I earnestly desire to see some systematic plan of control adopted for all these independent and now wholly irresponsible authorities, who have such important issues in their hands, and, as I have said before, in such control women must be allowed to take a part.

"X. Y. Z." writes: I am very glad to read the letter from "An Old Hand"; the subject is of such importance. A young woman begins her nursing work with the severe and usually unaccustomed physical strain of standing from nine to twelve hours daily. This alone is a hard matter to get over, but added to it there is in many a case the far harder mental strain of being perpetually harassed and worried by those over her. No one outside a hospital can have any idea of the manner in which probationers are spoken to, or the rudeness with which they have to put up. This, too, is not at any one hospital, but at many. Perhaps sisters do not consider teaching as part of their work, and so look upon it as a trouble and worry, which probationers must be made to feel as such, and be shown how ignorant and stupid they are. Whether the thing is done rightly or wrongly, they must be snubbed and their duties made as disagreeable as possible. Of course this is not always the case, but in the interest of the unfortunate victims who come under this type of upper nurse cannot something be done? Perhaps, as Miss Twining says, if women could be on the board of management a more thorough knowledge of the working of the teaching system as well as of the nursing might be had. A matron cannot see everything, but women in some official capacity, and yet outside the hospital system and etiquette, might be able to help in these ways. The instance "An Old Hand" gives of the rudeness of an upper nurse could be met by similar ones. Apart from this, Miss Twining suggests that the work should be gradual. What a relief that would be to many a beginner anxious to do well! Shop girls may sit occasionally; why not probationers? There is no rule in hospitals that nurses may not sit, but the actual fact is in most cases that a probationer found sitting down for a moment's rest would be called lazy and neglectful of her work. This is all of real importance, for what the probationer becomes the future sister will be; and any young woman trained under a system of rush, impatience, and inconsideration is apt, when her turn comes to rule, to use the same system with those under her.

### Novelties for Nurses.

### DRESSMAKING FOR NURSES.

ONE of the most convenient of modern inventions for the amateur dressmaker is Reddell's self-fitting dress bodice. It can be obtained at all linen-drapers for very little more than the cost of lining and bones alone. The self-fitting bodice renders what was an impossible task to many now comparatively simple. The bodice, which is of a really good cut, is nicely boned, and ready to be adapted to any figure and this can be done by the quite inexperienced amateur. Nurses who can devote a little time to needlework will be able to save a large amount of their earnings by making their own dresses, which the lining bodice renders possible.

### To Nurses.

**WE invite contributions from any of our readers, and shall be glad to pay for "Notes on News from the Nursing World," or for articles describing nursing experiences, or dealing with any nursing question from an original point of view. The minimum payment for contributions is 5s., but we welcome interesting contributions of a column, or a page, in length. It may be added that notices of entertainments, presentations, and deaths are not paid for, but, of course, we are always glad to receive them. All rejected manuscripts are returned in due course, and all payments for manuscripts used are made as early as possible at the beginning of each quarter.**

### For Reading to the Sick.

GOD'S DESIGN.

Joy and woe are woven fine,
A clothing for the soul divine;
Under every grief and pine
Runs a joy with silken twine.
It is right it should be so;
Man was made for joy and woe;
And, when this we rightly know,
Safely through the world we go.
*W. Blake.*

Not yet thou knowest how I bid
Each passing hour entwine
Its grief or joy, its hope or fear,
In one great love-design;
Nor how I lead thee through the night
By many a various way,
Still upward to unclouded light
And onward to the day. *F. R. H.*

One of the best aids to cheerfulness would be to put into words every little touch of pleasure, or gratitude, which lightens your load. We are all too much inclined to take these things silently, as a matter of course, and to put into words only those which annoy us. The world would be more full of sunshine if we made the contrary practice our rule of life. Do you know that excellent sermon in "Parables from Nature"—the story of the kitten that did not purr when he was pleased, and of the consequent depression in his family circle?

Do you keep silence from any unexpressed feeling that it is well for the happy busy lives around you to be reminded how long and dreary the day is to you? They are far more likely to awake to this when they hear you "purring" gratefully over some small pleasure, which they remorsefully feel would be as nothing to them. *When you count up your mercies, other people count up your crosses, and vice versâ.*

People talk as if sympathy were an ordinary straightforward virtue, like honesty—whereas it means either much experience or else the imagination of a genius combined with all the Christian graces of 1 Corinthians xiii.

People cannot understand things till they have experienced them. Try, therefore, not to chafe against blundering attempts to please you—against ignorant sympathy or ignorant want of sympathy. Let each such irritation remind you to thank God for the suffering which has made you of "a wise and understanding heart."—*L. H. M. Soulsby.*

There are who sigh that no fond heart is theirs,
None loves them best!—O vain and selfish sigh!
Out of the bosom of His love, He spares—
The Father spares the Son,—for thee to die:
For thee He died, for thee He lives again;
O'er thee He watches in His boundless reign.

Thou art as much His care, as if beside
Nor man nor angel lived in Heav'n or earth:
Thus sunbeams pour alike their glorious tide,
To light up worlds, or wake an insect's mirth;
They shine and shine with unexhausted store—
Thou art Thy Saviour's darling—seek no more!—*Keble.*

### Notes and Queries.

**The Editor is always willing to answer in this column, without any fee, all reasonable questions, as soon as possible.**
**But the following rules must be carefully observed:—**
1. **Every communication must be accompanied by the name and address of the writer.**
2. **The question must always bear upon nursing, directly or indirectly.**

**If an answer is required by letter a fee of half-a-crown must be enclosed with the note containing the inquiry.**

*Infectious Diseases.*

(209) Will you kindly inform M. L. B. if there is a home hospital in London where young children of the upper classes suffering from infectious disorders can be received, accompanied by a nurse.

There is no institution especially constituted to receive cases on such terms, but arrangements might be made with the London Fever Hospital, Liverpool Road, Islington, or your medical attendant could doubtless make similar arrangements privately with some one or other of the public infectious disease hospitals. The Mary Wardell Home, Stanmore, Middlesex, receives convalescents from scarlet fever on those terms.

*Young Probationer.*

(210) I must do something for myself, and should like to begin as soon as possible. Are there any hospitals in Scotland or England which a probationer of eighteen could enter, with or without salary?—*Flossie.*

Could you not go as mother's help, or assistant to a lady? It is not worth while to attempt to train before twenty-one, and meanwhile you could get remunerative work and learn much in domestic employments.

*Germany.*

(211) Can you tell me what hospitals in Germany give good general training to nurses? How long does the training last, and is any salary given? Would a nurse holding a certificate from a German hospital be able to obtain work in a public or private institution in England?—*Ria.*

An article on "Nursing in North Germany" was published in No. 732 of Nursing Mirror, dated October 6th, 1900, which will probably be of use to you. The system of training on the Continent is so widely different from that obtaining in this country that it would be wiser to gain an English qualification if you intend working here.

*R. B. N. A.*

(212) Will you kindly tell me how an individual nurse benefits by becoming a member of the Royal British Nurses' Association, and what is the object of the society collectively.—*E. M. D.*

The individual nurse has the benefit of experienced counsel in cases of difficulty and distress. The aim of the Association is to band qualified nurses into a union for mutual help and improvement. Apply the Secretary, 17 Old Cavendish Street, W., for full information.

*Hospital Prayers.*

(213) Can you recommend a book of prayers for daily use in the wards of a cottage hospital? There are frequently children present.—*Matron.*

Many matrons compile selections from devotional books for their own use. Perhaps some of our readers can mention a book they have found helpful.

*Children's Hospital.*

(214) Is there a good children's hospital in Lancashire where probationers are taken at the age of 20 or under?—*L. R.*

Probationers of 20 are taken at the Wirral Hospital and Dispensary for Sick Children, Woodchurch Road, Birkenhead. Birkenhead, though in Cheshire, is practically part of Liverpool.

*India.*

(215) Would the editor inform Nurse E. H. where she ought to apply for information about nursing in India under Government?

Information respecting the Indian Nursing Service is supplied by the India Office, St. James's Park, S.W.

*Maternity Training.*

(216) Kindly tell me which is the best, the L.O.S. or the Midwifery? I am thinking of taking up monthly work, and have been told that the training given by the Plaistow Maternity Charity is very good.—*A. B. C.*

The L.O.S. is the London Obstetrical Society, which awards candidates who have passed a satisfactory examination in midwifery a certificate of the fact. You can be prepared for the examination at Plaistow or at any of the lying-in hospitals. The hospitals, however, have greater teaching facilities than the Maternity Charity.

*Australia.*

(217) Kindly tell me where I can obtain particulars of nursing appointments in Western Australia, New South Wales, or New Zealand. Can you inform me if a trained nurse holding L.O.S. certificate would have much difficulty in obtaining a post in any of these countries?—*E. S.*

Nurses are not encouraged to go out to Australasia because the local supply of nurses is fully equal to the demand. Apply for latest returns as to prospects of work to the Emigrants' Information Office, 31 The Broadway, Westminster, S.W. Appointments can be obtained only on the spot.

*Dispensing.*

(218) Can you give me any information as to how to become a lady dispenser, how to train, and the shortest time in which I could train?—*I. N. M.*

Write to the secretary of the Pharmaceutical Society, 77 Bloomsbury Square, S.W, for answer to your question

**Standard Books of Reference.**

"The Nursing Profession: How and Where to Train." 2s. net; post free, 2s. 4d.
"The Nurses' Dictionary of Medical Terms." 2s.
"Burdett's Series of Nursing Text-Books." 1s. each.
"A Handbook for Nurses." (Illustrated.) 5s.
"Nursing: Its Theory and Practice." New Edition. 3s. 6d.
"Helps in Sickness and to Health." Fifteenth Thousand.
"The Physiological Feeding of Infants." 1s.
"The Physiological Nursery Chart." 1s.; post free, 1s. 3d.
"Hospital Expenditure: The Commissariat." 2s. 6d.

All these are published by The Scientific Press, Ltd., and may be obtained through any bookseller or direct from the publishers, 28 and 29 Southampton Street, London, W.C.

